# Early Local Tumour Control and Feasibility of Intratumoral P-32 Microparticle Implantation in Patients with Locally Advanced Pancreatic Cancer: A Prospective Single-Center Observational Cohort Study

**DOI:** 10.3390/cancers18203331

**Published:** 2026-10-15

**Authors:** Athanasios Kontos, Ioannis Riris, Dimitrios Kypraios, Nikolaos Alexakis, Georgios Zografos, Konstantinos Toutouzas

**Affiliations:** 1 Endoscopy Unit, Gastroenterology Department, Hospital Universitario Río Hortega, 47012 Valladolid, Spain; 2 Medical School of Athens, National and Kapodistrian University of Athens, 11527 Athens, Greece; 3 Gastroenterology Department, Agios Savvas Hospital, 11522 Athens, Greece; 4 First Propaedeutic Department of Surgery, School of Medicine, National and Kapodistrian University of Athens, Hippocration General Hospital of Athens, 11527 Athens, Greece

**Keywords:** pancreatic cancer, intratumoral therapy, radioactive microparticles, local tumour control, phosphorus-32, brachytherapy, surgical resection

## Abstract

**Background**: Evidence regarding EUS-guided intratumoral phosphorus-32 (P-32) microparticle implantation for locally advanced pancreatic cancer (LAPC) remains limited. This prospective pilot study evaluated its technical feasibility, preliminary safety, and early disease-control outcomes when administered in combination with gemcitabine plus nab-paclitaxel. **Methods**: Seventeen patients with unresectable LAPC underwent EUS-guided intratumoral P-32 microparticle implantation through a 22-gauge fine-needle aspiration needle during the fourth week of systemic chemotherapy. Intratumoral distribution was assessed using Bremsstrahlung SPECT/CT. Tumour response was evaluated according to RECIST version 1.1, and treatment-emergent adverse events were graded according to CTCAE version 4.0. The main secondary endpoint was local disease control rate at approximately 16 weeks (LDCR16w). All analyses were descriptive. **Results**: Implantation was technically successful in all 17 patients (100.0%; exact 95% CI, 80.5–100.0%), and SPECT/CT confirmed intratumoral distribution in every case. No implantation-related procedural complications were recorded (0/17; exact 95% CI, 0.0–19.5%). At approximately 16 weeks, CT assessment was available for 16 patients: seven had a partial response and nine had stable disease. The LDCR16w was 16/16 among evaluable patients (100.0%; exact 95% CI, 79.4–100.0%) and 16/17 in the all-treated analysis (94.1%; exact 95% CI, 71.3–99.9%). A reduction in CA19-9 of at least 50% was observed in 13/16 evaluable patients. Four patients underwent subsequent R0 resection (4/17, 23.5%; exact 95% CI, 6.8–49.9%). Adverse events were predominantly grade 1–2; one grade 3 abdominal pain event and no grade 4–5 events were recorded. Only three deaths occurred during a median OS follow-up of 15.6 months, making survival estimates exploratory and statistically immature. **Conclusions**: In this small, single-center pilot cohort of highly selected patients, EUS-guided intratumoral P-32 microparticle implantation combined with gemcitabine plus nab-paclitaxel was technically feasible and provided preliminary safety information. Because of the absence of a chemotherapy-only comparator, the small sample size, and the limited follow-up, the radiographic, biomarker, resection, and survival outcomes cannot be attributed specifically to P-32 implantation and should be considered descriptive and hypothesis-generating. Larger controlled studies with longer follow-up are required to determine clinical efficacy.

## Simple Summary

Locally advanced pancreatic cancer is difficult to treat and cannot usually be removed surgically at diagnosis. This pilot study evaluated whether radioactive phosphorus-32 (P-32) microparticles could be safely and successfully delivered directly into pancreatic tumours under endoscopic ultrasound guidance, together with standard chemotherapy. Seventeen patients received the combined treatment. Implantation was technically successful in all patients, and no procedure-related complications were recorded. Early imaging showed partial tumour shrinkage or stable disease in the 16 evaluable patients, while four patients subsequently underwent surgery. However, because the study included only 17 highly selected patients and did not have a chemotherapy-only control group, these outcomes cannot be attributed specifically to P-32 treatment. The findings mainly demonstrate technical feasibility and provide preliminary safety information. Larger controlled studies with longer follow-up are needed to determine whether P-32 implantation improves tumour control, surgical resection rates, or survival.

## 1. Introduction

Pancreatic cancer is the seventh most common cancer and the fourth cancer-related cause of death in the Western world [1]. Although there have been advances in the treatment of pancreatic cancer, mortality remains high [2].

A substantial proportion of patients (20–30%) present with locally advanced pancreatic cancer (LAPC), in which uncontrolled local tumour progression contributes significantly to morbidity and precludes potentially curative surgical resection [3,4]. While modern multi-agent chemotherapy regimens have improved systemic disease control, durable local tumour control remains a major clinical challenge due to the chemoresistance of pancreatic cancer [5]. Unresectable LAPC has a poor prognosis, with a median overall survival (OS) of approximately 13.3 months, although some studies have reported survival up to 19.9 months [6].

Standard systemic treatment options for LAPC include FOLFIRINOX and gemcitabine plus nab-paclitaxel. The combination of systemic chemotherapy with radiotherapy may improve local disease control, pain management, and quality of life and may increase the likelihood of tumour resection [6,7,8,9,10]. However, the improvement in OS is limited [10,11,12,13]. In addition, radiation therapy may cause damage to surrounding organs, highlighting the need for more precise radiation techniques [6].

Locoregional treatment strategies have therefore gained increasing attention as potential adjuncts to systemic therapy [4]. These approaches aim to enhance local tumour control while limiting toxicity to surrounding organs. Intratumoral brachytherapy using radioactive microparticles represents one such strategy, enabling delivery of high radiation doses directly to the tumour [14,15,16].

Among patients with locally advanced pancreatic cancer (LAPC), intratumoural brachytherapy using radioactive isotopes has emerged as a promising therapeutic strategy. By delivering a high radiation dose directly to the tumour while limiting irradiation of the surrounding normal tissues, this approach may enhance local tumour control with reduced off-target toxicity. The radioactive agents most extensively investigated to date are iodine-125 (125I) seeds and P-32 microparticles, whereas clinical experience with other isotopes, including iodine-131 (131I) and yttrium-90 (90Y), remains limited. Additional radionuclides that have been explored for pancreatic cancer brachytherapy include holmium-166 poly-L-lactic acid microspheres (166HoMS), palladium-103 (103Pd), yttrium-90 (90Y), and iridium-192 (192Ir) for which clinical experience and outcome data also remain limited [17,18].

Radioactive silicon-based microparticles incorporating beta-emitting P-32 isotopes are a novel brachytherapy device developed for direct intratumoral implantation through endoscopic ultrasound (EUS). The maximum range of emission of each microparticle is approximately 8 mm and the average is 2.76 mm. The mean physical half-life is 14.27 days. The device is intended for use in unresectable LAPC and is administered to deliver an absorbed dose of 100 Gy to the tumour. In therapeutic use, 98% of the radiation is delivered within 81 days [19]. Chemotherapy was not administered for 48 h before or after P-32 implantation, in accordance with the instructions for use of P-32. Clinical studies and early clinical experience suggest that this approach may achieve meaningful local tumour control with an acceptable safety profile [20,21,22]. However, clinical evidence remains limited, and real-world data describing early radiographic response, feasibility, and safety are scarce.

The present study reports early outcomes from a prospective single-center observational cohort study of patients treated with intratumoral radioactive microparticle implantation, focusing on local tumour response, local disease control, safety, and subsequent surgical feasibility.

## 2. Methods

### 2.1. Study Design and Patients

This was a prospective single-center observational cohort study of consecutive patients with locally advanced pancreatic cancer, who received intratumoral P-32 microparticle implantation in combination with gemcitabine plus nab-paclitaxel at Agios Savvas Hospital between December 2023 and June 2025. Follow-up data were available through 23 January 2026. The analysis was prospective and exploratory in nature.

Adult patients with histologically or cytologically confirmed unresectable LAPC were eligible for inclusion. Patients were classified as having unresectable LAPC based on multiphasic contrast-enhanced CT findings, according to NCCN/ESMO criteria. Unresectability was defined by relevant arterial contact >180°, unreconstructible venous involvement, in the absence of distant metastatic disease. The imaging findings and treatment strategy were reviewed by a multidisciplinary tumour board comprising radiologists, surgeons, oncologists and gastroenterologists. Additional eligibility criteria included a tumour diameter of 2–7 cm, an Eastern Cooperative Oncology Group (ECOG) performance status of 0 or 1, adequate renal, hepatic, and bone marrow function, and absence of pregnancy. All the above eligibility criteria are specified in the Instructions for Use of radioactive phosphorus-32 (P-32).

The exclusion criteria were evidence of metastatic pancreatic cancer on computed tomography (CT); resectable or borderline resectable pancreatic cancer on CT; radiographic or endoscopic evidence of invasion of the stomach, duodenum, or peritoneum; the presence of more than one primary tumour; previous treatment for pancreatic cancer before study enrolment; participation in another investigational study; a history of another malignancy; known hypersensitivity to silicon or phosphorus; and technically infeasible or unsafe EUS-guided implantation, as determined by the investigator. Implantation was considered technically infeasible or unsafe when a stable transgastric or transduodenal needle trajectory into the tumour could not be established; when intervening vessels, the pancreatic or bile duct, or adjacent hollow viscera could not be safely avoided; when the lesion could not be adequately visualized or accessed by EUS; or when the anticipated needle position would not permit reliable intratumoral delivery without an unacceptable risk of off-target implantation.

### 2.2. Treatment Procedure

The included patients received P-32 microparticles (OncosilTM Medical, Sydney, Australia) and chemotherapy with gemcitabine plus nab-paclitaxel. The implantation was performed during the fourth week after the initiation of chemotherapy, in accordance with the manufacturer’s Instructions for Use. The chemotherapy was withheld for 48 h before and after implantation. The activity of P-32 was calculated using the patient’s tumour volume in order to deliver a 100 Gy absorbed dose. The administered activity was recorded procedurally; however, quantitative post-implantation measurement of retained intratumoral activity was not performed.

Radioactive P-32 loaded microparticles were implanted directly into the pancreatic tumour via EUS guidance using a 22-gauge fine-needle aspiration needle. Technical success of implantation was confirmed by post-procedural Bremsstrahlung single-photon emission CT (SPECT/CT) within 4 h and at 24 h of implantation. The integration of nuclear medicine with complementary imaging modalities is increasingly relevant to precision oncology because it can combine functional or molecular information with anatomic localization [23]. In the context of intratumoral P-32 implantation, Bremsstrahlung SPECT/CT enables post-procedural visualization of radionuclide distribution relative to tumour anatomy.

### 2.3. Tumour Assessment and Response Definitions

Patients were evaluated on the day before chemotherapy initiation and weekly thereafter until week 16. These assessments included clinical examination, ECOG performance status, symptom evaluation, adverse-event recording, and haematological and biochemical laboratory tests, including CA19-9. Subsequently, contrast-enhanced CT of the abdomen and laboratory tests, including CA19-9 measurements, were performed every 8 weeks during the first year after implantation and every 12 weeks thereafter until death or completion of two years of follow-up.

In addition to staging and response assessment, multiphase contrast-enhanced CT contributes to pancreatic tumour characterization; recent work has identified features such as tumour margin, pancreatic duct interruption, and pancreatic parenchymal-phase attenuation as potentially useful in differentiating pancreatic acinar cell carcinoma from pancreatic ductal adenocarcinoma [24].

Tumour marker carbohydrate antigen 19-9 (CA19-9) was measured at baseline and at each scheduled follow-up time point. Among patients with a baseline CA19-9 level >37 U/mL and an available 16-week measurement, a reduction ≥50% from baseline values was considered clinically important, if baseline levels were >37 U/ml. CA 19-9 levels before treatment and during follow-up were assessed only in the absence of biliary obstruction or other factors that could confound the measurements.

### 2.4. Primary and Secondary Endpoints

The primary endpoint was safety, feasibility, and tolerability, assessed using treatment-emergent adverse events (TEAEs) graded according to the National Cancer Institute Common Terminology Criteria for Adverse Events (CTCAE), version 4.0, from the date of implantation until the end of the study. TEAEs and laboratory values were assessed at each visit until the end of study. Procedural complications related to implantation were routinely documented in procedural reports and medical records.

The main secondary endpoint was local disease control rate at approximately 16 weeks (LDCR16w), defined as the proportion of patients with CR, PR, or SD at the scheduled 4-month CT assessment after implantation. The primary LDCR16w analysis included patients with an available CT assessment at approximately 16 weeks. For the complementary all-treated analysis, all 17 treated patients were included in the denominator; the patient without an evaluable 16-week CT assessment did not contribute to the numerator. Tumour size was assessed by contrast-enhanced computed tomography (CT) at baseline and at scheduled follow-up time points using RECIST version 1.1 [25], independently assessed by radiologists. According to RECIST version 1.1, complete response (CR) was defined as the disappearance of all target lesions; partial response (PR) as a decrease of at least 30% in the sum of target-lesion diameters, using the baseline sum as reference; progressive disease (PD) as an increase of at least 20% in the sum of target-lesion diameters, using the smallest recorded sum as reference, with an absolute increase of at least 5 mm; and stable disease (SD) as neither sufficient tumour shrinkage to qualify as PR nor sufficient increase to qualify as PD.

Other secondary endpoints included percent change in tumour diameter over time, distribution of response categories, surgical resection rate following treatment, and exploratory time-to-event outcomes, including local progression-free survival (LPFS), CA19-9 variations and overall survival (OS). LPFS was defined as the time of initiation of systemic chemotherapy to the date of the scan that confirmed local tumour progression. OS was defined as the time from initiation of systemic chemotherapy to death by any cause.

### 2.5. Statistical Analysis

All analyses were descriptive. No formal sample-size calculation was performed because of the exploratory and descriptive nature of the study; all consecutive eligible patients treated during the predefined enrolment period were included. Continuous variables were summarized using means, standard deviations, medians, interquartile ranges. Categorical variables were summarized using counts and percentages, with exact binomial 95% confidence intervals (Clopper-Pearson) for proportions.

Tumour response at 16 weeks was summarized from available target-lesion diameter measurements, and waterfall plotting was used to visualize percentage change in tumour diameter. Time-to-event outcomes, including LPFS and OS, were estimated using Kaplan–Meier methods and interpreted as exploratory because of the small sample size and limited number of events. Time-to-event outcomes were estimated from both initiation of systemic chemotherapy and the date of P-32 implantation. The treatment-initiation analyses describe outcomes from the beginning of the combined treatment pathway, whereas the implantation-based analyses provide a time origin aligned with exposure to P-32 and avoid attributing the pre-implantation treatment period to P-32. All time-to-event analyses were considered exploratory. A sensitivity analysis was performed to assess the potential effect of surgical resection on LPFS estimates. In this analysis, patients who underwent surgical resection without prior local progression were censored at the date of their last available CT assessment before surgery. No formal hypothesis testing was performed due to the small sample size and exploratory nature of the study.

## 3. Results

### 3.1. Patient and Treatment Characteristics

Seventeen patients were included in the efficacy and safety analyses. The median age was 70 years (IQR 60–75). 11 patients (64.7%) were male and 6 patients were female (35.3%). ECOG performance status was 0 in 10 patients (58.8%) and 1 in 7 patients (41.2%). In 11 patients, the primary tumour was located in the pancreatic head (64.7%), followed by the body in 4 (23.5%), the neck in 1 (5.9%), and the uncinate process in 1 (5.9%). Median tumour volume was 20.0 cc (IQR 11.6–29.0). Regarding treatment delivery, the median P-32 microparticles solution volume was 1.6 mL (IQR 0.9–2.3), and the median administered activity was 10.5 MBq (IQR 6.1–15.2). Baseline characteristics of the population are summarised in Table 1.

### 3.2. Procedural Feasibility

Intratumoral implantation was technically successful in all 17 patients. SPECT/CT imaging confirmed appropriate orthotopic intratumoral distribution in every case (17/17, 100.0%; exact 95% CI 80.5–100.0%). No implantation-related complications were recorded (0/17, 0.0%; exact 95% CI 0.0–19.5%). Procedural feasibility was assessed by the endoscopist immediately after the procedure and was considered technically successful. Following P-32 implantation, all patients continued chemotherapy without interruption according to the standard treatment schedule.

During implantation, the only technical difficulty encountered was increased backpressure, which markedly impeded the flow of the microparticle solution into the tumour. This issue was encountered in 14 of the 17 patient was readily resolved by slight repositioning of the needle within the tumor, most commonly by minimal withdrawal of the needle without removing it from the lesion.

### 3.3. Tumour Size Dynamics and Local Response

**Figure 1:**
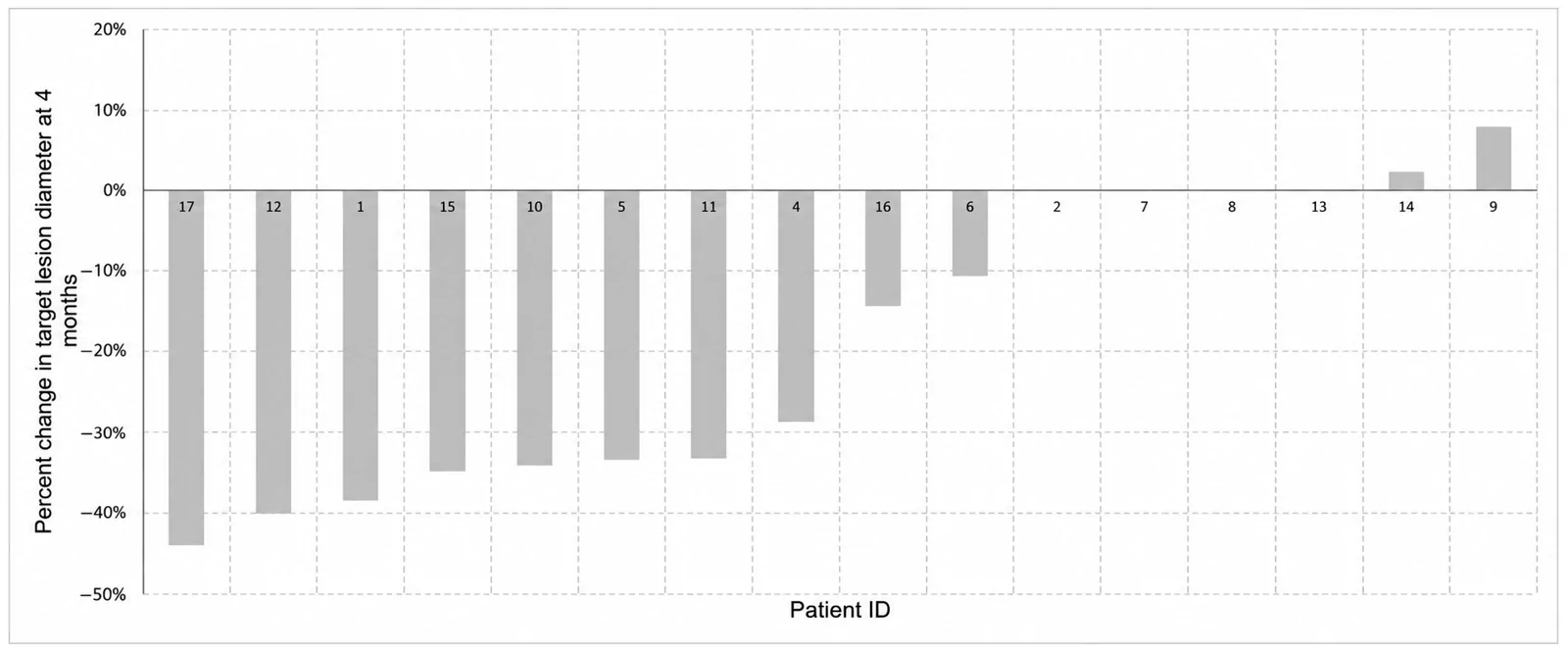
Waterfall plot of percentage change in target-lesion diameter at approximately 16 weeks among evaluable patients (n = 16).

Baseline mean tumour diameter was 3.4 cm, with a median of 3.0 cm (IQR 2.5–3.9). At 16 weeks, the mean diameter decreased to 2.8 cm among participants, with a median of 2.8 cm (IQR 2.5–3.0). The median percentage change was −21.4% (IQR −34.4 to 0.0) and the median absolute diameter reduction was −0.6 cm (IQR −1.3 to 0.0). Individual percentage changes in target-lesion diameter at 16 weeks are shown in Figure 1.

**Figure 2:**
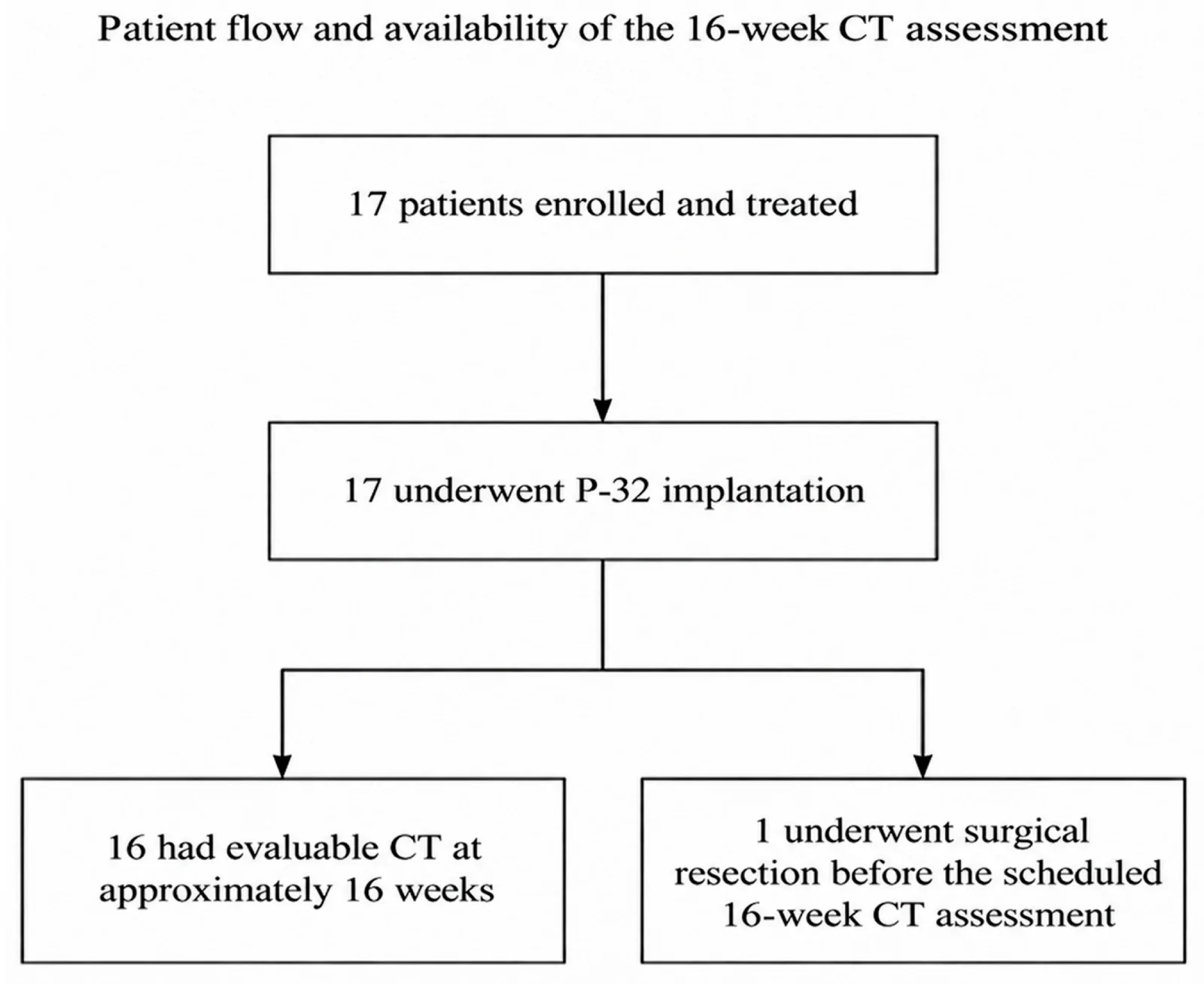
Patients flow and availability at 16 weeks assesement.

CT assessment at approximately 16 weeks was available for 16 of 17 patients, as one patient underwent surgery before reaching the 16-week post-implantation time point. Based on available target diameter measurements, partial response was observed in 7 out of 16 patients (43.8%; exact 95% CI 19.8–70.1) and stable disease in 9 out of 16 (56.2%; exact 95% CI 29.9–80.2). No cases of early local progression or complete response were observed. The LDCR16w was therefore 16/16 (100.0%; exact 95% CI 79.4–100.0) among evaluable patients and 16/17 (94.1%; exact 95% CI 71.3–99.9), using all 17 treated patients as the denominator. These findings represent descriptive early disease control and should not be interpreted as evidence of treatment efficacy. Patient disposition and availability of the 16-week radiological assessment are summarized in Figure 2.

### 3.4. CA19-9 Response

CA19-9 levels decreased during early follow-up. At 16 weeks, 13 out of 16 patients (81.2%; exact 95% CI, 54.4–96.0) showed a reduction of at least 50% from baseline, while the median percentage change in CA19-9 was −73.7% (IQR, −89.6% to −52.4%).

### 3.5. Surgical Resection

Four patients (4/17, 23.5%; exact 95% CI 6.8–49.9) underwent surgical resection following treatment. Three of the four resected patients had demonstrated a partial response at the 4-month assessment, whereas the fourth patient underwent surgery before the 4-month post-implantation assessment after demonstrating a partial response at the 2-month follow-up. All four resections achieved R0 status, according to postoperative pathological assessment.

The decision to proceed to surgery was made by multidisciplinary tumour board after reassessment of vascular involvement, absence of metastatic progression and performance status.

### 3.6. Safety

Adverse events were common after implantation but predominantly low-grade, as reported by the patients and assessed by the investigators according to the Common Terminology Criteria for Adverse Events (CTCAE). Any adverse event was recorded in 12/17 patients (70.6%; exact 95% CI 44.0–89.7). Nausea occurred in 9 out of 17 patients (52.9%), abdominal pain in 8 out of 17 (47.1%) and fatigue in 7 out of 17 (41.2%). Most events were grade 1–2, one grade 3 abdominal pain event was recorded (1/17, 5.9%), and no grade 4–5 adverse events were recorded. No implantation-related procedural complications were recorded.

### 3.7. Follow-Up and Time-to-Event Outcomes

Time-to-event outcomes were evaluated descriptively using Kaplan–Meier analysis because of the limited sample size. Survival outcomes were assessed from both treatment initiation and the date of P-32 implantation, as the P-32 implantation was performed after the systemic treatment’s initiation.

**Figure 3:**
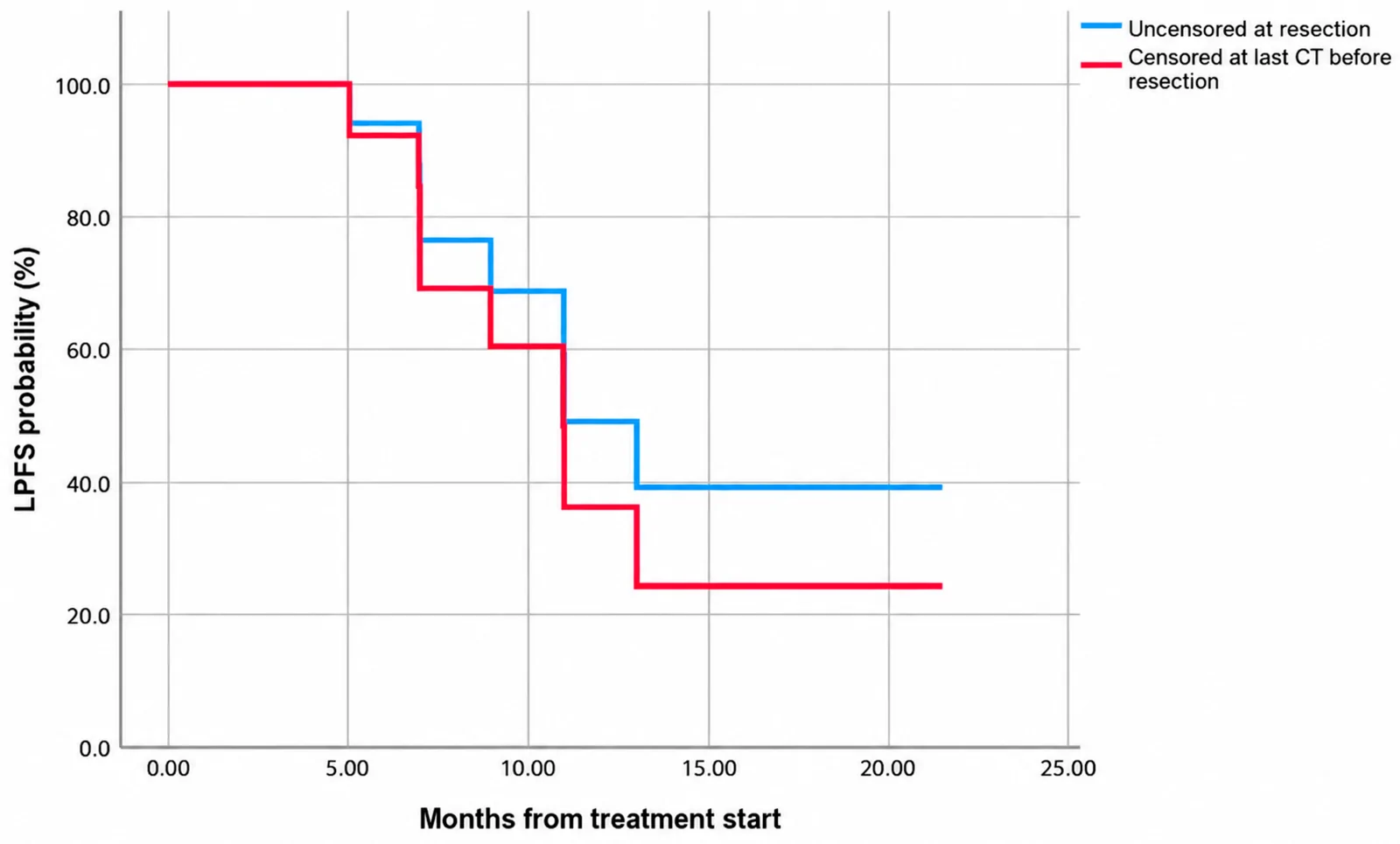
Kaplan–Meier curve of local progression-free survival from treatment initiation.

**Figure 4:**
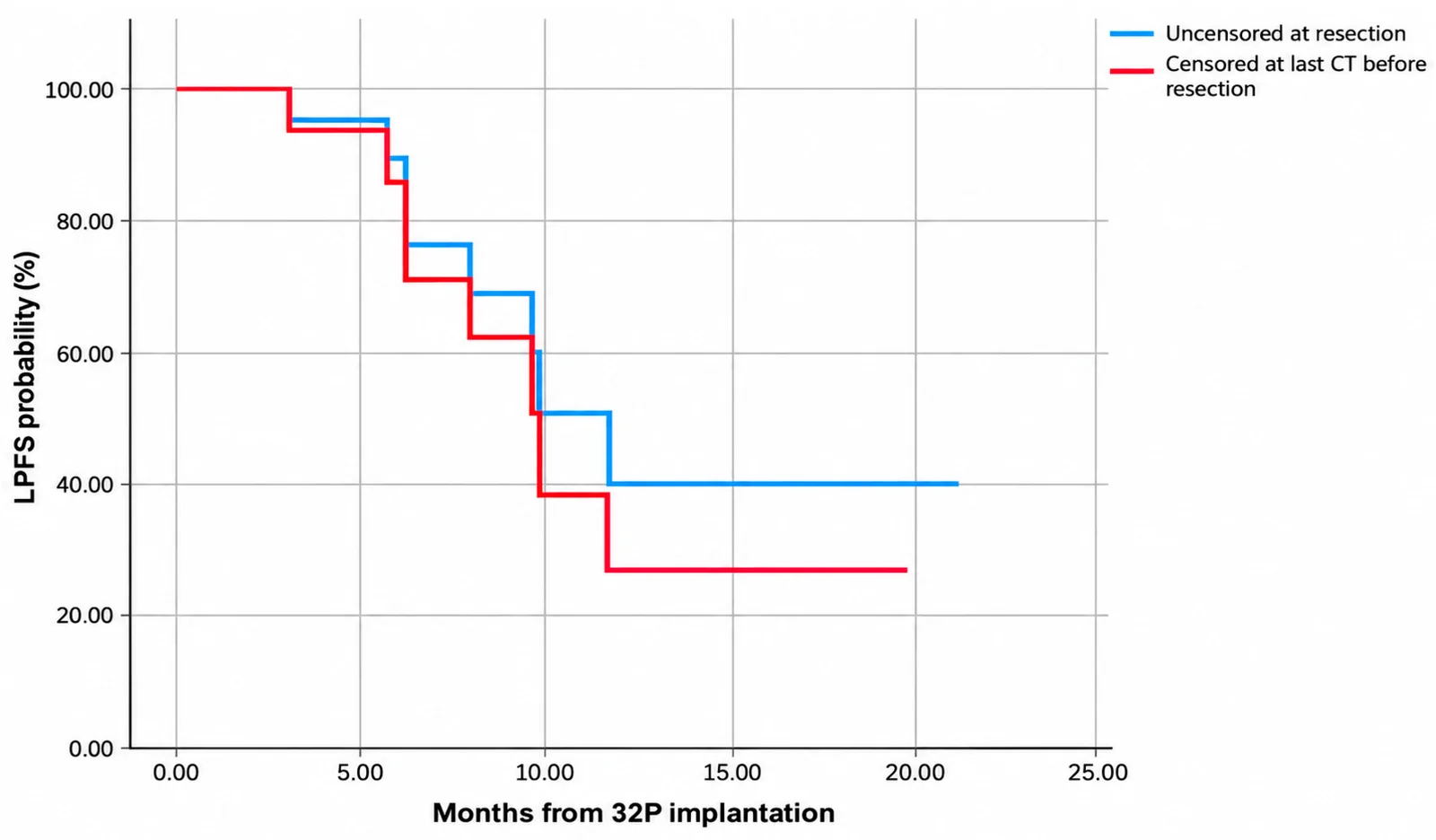
Kaplan–Meier curve of local progression-free survival from P-32 implantation.

Median LPFS was 11.0 months (Figure 3) from initiation of systemic chemotherapy and 9.7 months (Figure 4) from P-32 implantation. To account for the potential effect of subsequent surgical resection on LPFS estimation, an additional sensitivity analysis was performed. In this analysis patients who underwent surgical resection without prior local progression were censored at the last available CT scan before surgery. The median LPFS was 11.0 months from treatment initiation and 9.6 months from the date of P-32 implantation.

**Figure 5:**
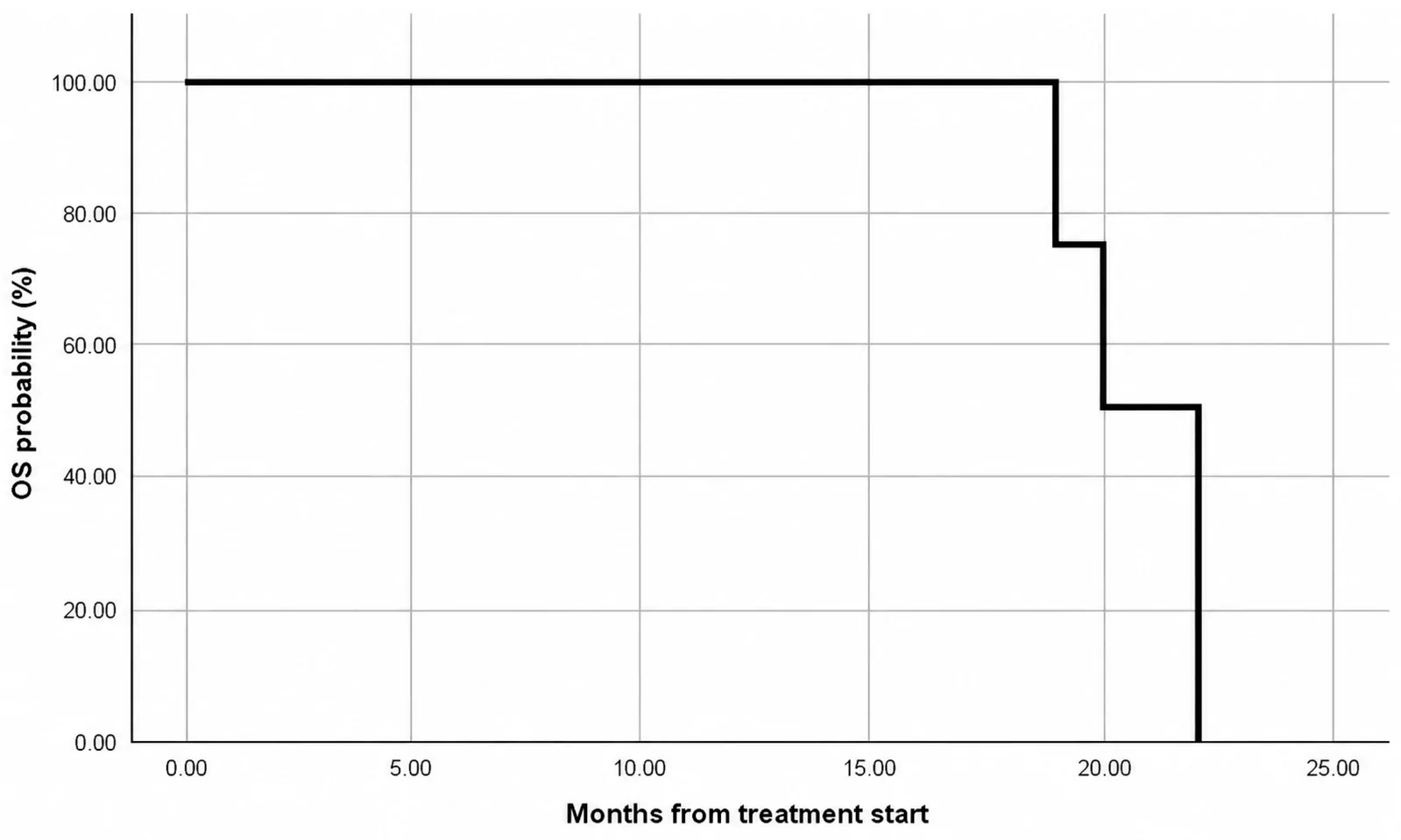
Kaplan–Meier analysis of overall survival from treatment initiation.

**Figure 6:**
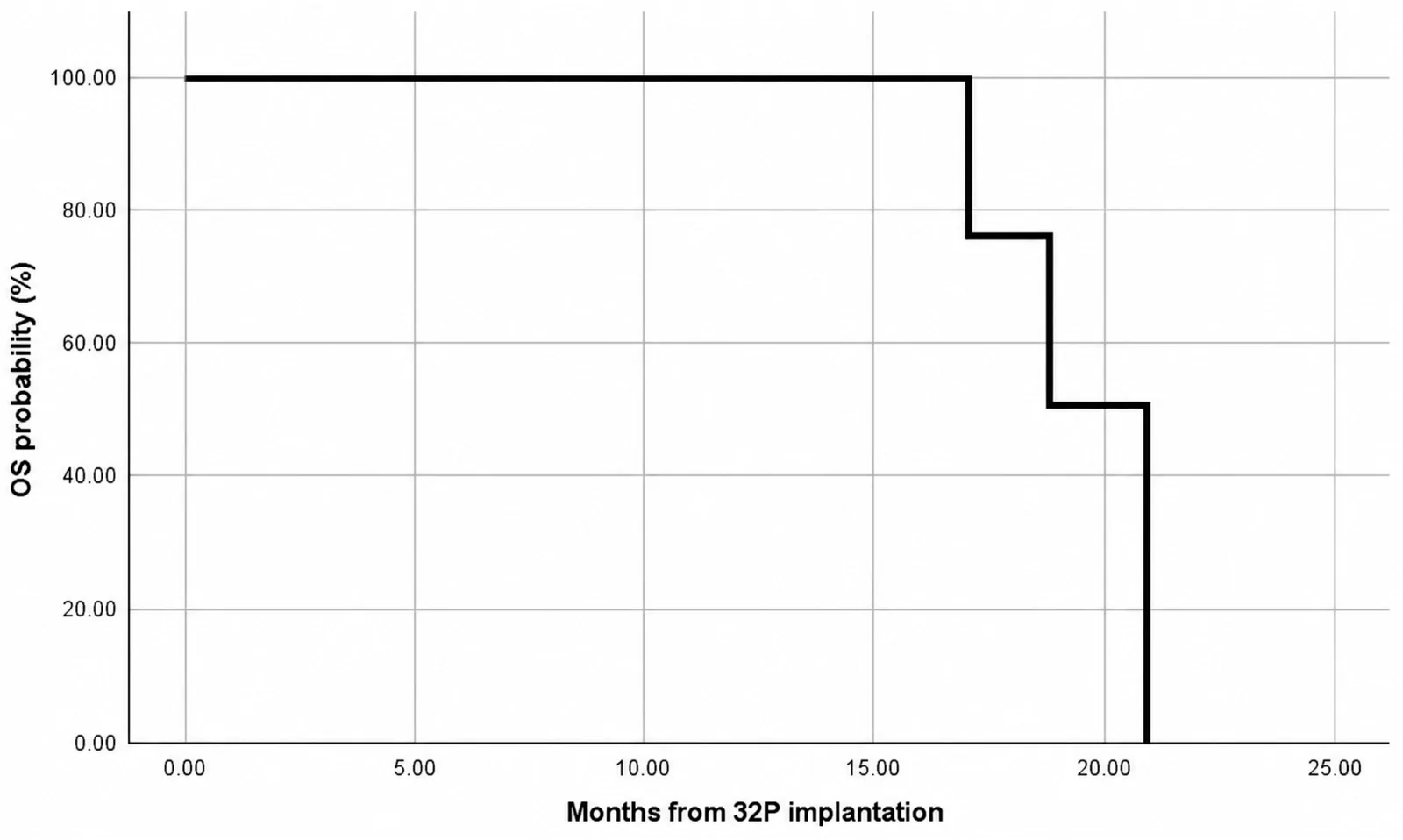
Kaplan–Meier analysis of overall survival from P-32 implantation.

The estimated median OS was 20.0 months when calculated from treatment initiation (Figure 5) and 18.8 months when calculated from P-32 implantation (Figure 6). These estimates should be interpreted with substantial caution because only three deaths had occurred at the time of analysis, resulting in statistically immature and imprecise survival estimates. The median follow-up from treatment initiation was 15.6 months for OS and 10.7 months for LPFS.

## 4. Discussion

In this prospective single-center observational cohort study, intratumoral implantation of P-32 microparticles was technically feasible and demonstrated an acceptable safety profile when combined with gemcitabine plus nab-paclitaxel in patients with LAPC. This study reports preliminary outcomes from a prospective observational cohort, focusing on procedural feasibility, early local tumour control, CA19-9 response, safety, and subsequent surgical resection.

Previous evidence suggests that, even in the presence of micrometastatic disease, improved local tumour control may contribute to better symptom control and quality of life [22]. Accordingly, locoregional tumour control remains an important therapeutic objective. At 16 weeks following the implantation, all evaluable patients demonstrated local disease control, with nearly half achieving partial radiographic tumour shrinkage and no cases of early local progression observed.

The observed reduction in tumour diameter, high local disease control rate at 16 weeks, and decline in CA19-9 levels describe early tumour-control outcomes following the combined treatment strategy of systemic chemotherapy and intratumoral P-32 implantation. Given the absence of a chemotherapy-only comparator, the independent contribution of P-32 cannot be determined. Local disease progression is a major contributor to morbidity and impaired quality of life in pancreatic cancer [26,27,28]. In a prospective cohort spanning all disease stages, Crippa et al. demonstrated that patients with locally advanced disease experienced persistent pain, biliary and gastrointestinal obstruction, and limited improvements in health-related quality of life despite systemic and locoregional treatments, whereas meaningful improvements were largely confined to patients undergoing surgical resection [29]. In this context, strategies that achieve early local control may contribute to symptom palliation and, in selected patients, enable subsequent surgical resection. Four of seventeen patients subsequently underwent surgical resection; however, given the small cohort and absence of a comparator group, this observation should not be interpreted as evidence that P-32 increased the probability of conversion to resection. Notably, all resected patients achieved R0 status on postoperative pathological assessment.

Previous studies have proposed potential biological interactions between intratumoral irradiation and systemic chemotherapy, including changes in tumour vascularity that may influence chemotherapy delivery [30]. However, the present study was not designed to assess treatment synergy, and the absence of a chemotherapy-only comparator precludes any conclusion regarding a synergistic effect or the independent contribution of P-32 implantation. Patients with locally advanced pancreatic cancer (LAPC) who undergo surgical resection experience substantially longer overall survival (OS) than those who remain unresectable (median OS, 35.3 vs. 16.3 months) [31]. In our survival outcomes should be interpreted cautiously because only three deaths had occurred at the time of analysis. Accordingly, the median OS estimates are statistically immature and exploratory and should not be interpreted as evidence of a survival benefit associated with P-32 implantation. Furthermore, achieving an R0 resection has been associated with significantly improved OS compared with an R1 resection [31,32].

The results observed in our selected real-world population, which met the device-specific eligibility criteria, are broadly consistent with previously published prospective evidence [22,29,30,31]. The PanCO multicentre pilot study evaluated intratumoral P-32 microparticles in combination with systemic chemotherapy in patients with unresectable locally advanced pancreatic cancer and reported high early local disease control, acceptable safety, and a resection rate of approximately 24% in the per-protocol population [22]. The study reported an LDCR at week 16 of 82.0% in the intention-to-treat population and 90.5% in the implanted per-protocol population. While the PanCO study differed substantially from the present analysis in terms of scale, prospective design and multicentre conduct, the direction and magnitude of early local control observed in our cohort appear concordant. Importantly, these similarities should be interpreted cautiously and do not imply equivalence or superiority. Rather, they demonstrate that similar early disease-control outcomes have been observed in other cohorts receiving P-32 implantation in combination with systemic chemotherapy; however, these uncontrolled comparisons do not establish treatment efficacy or causality. Notably, all patients in our cohort received the same chemotherapy regimen.

Another prospective single-center trial by A.H.W. Lim et al. demonstrated that, in patients with unresectable locally advanced pancreatic cancer (LAPC) treated with chemotherapy combined with P-32 microparticles, tumour vascularity increased, potentially enhancing chemotherapy delivery to cancer cells and thereby improving its efficacy. Nevertheless, the underlying mechanism responsible for this effect remains unclear. That study reported a technical success rate of 100%, a resection rate of 25%, a local disease control rate (LDCR) at 12 weeks of 100%, and a median tumour reduction of 8 mm at 12 weeks [30].

Safety outcomes in the present cohort were acceptable. No procedural implantation-related complications were recorded. Technical feasibility was 100%, with the endoscopist reporting relatively easy implantation in all procedures. Adverse events were predominantly grade 1–2, although one grade 3 abdominal pain event was observed. No grade 4–5 adverse events were recorded. Overall, the safety profile aligns with the concept of localized radiation delivery achieving tumour-directed effects while limiting systemic toxicity.

This study has several major limitations. First, the small sample size (n = 17) and single-center design result in imprecise estimates with wide confidence intervals and make the findings vulnerable to random variation and selection effects. In addition, the device-specific eligibility criteria selected patients with ECOG performance status 0–1, tumour diameter of 2–7 cm, adequate organ function, absence of metastatic or adjacent-organ disease, and anatomy suitable for safe EUS-guided implantation. The cohort therefore represents a highly selected population, limiting the generalizability of the findings to the broader population of patients with LAPC. Second, all patients received P-32 implantation in combination with gemcitabine plus nab-paclitaxel, without a chemotherapy-only comparator. Consequently, the observed tumour shrinkage, CA19-9 reduction, local disease control, and subsequent resections cannot be attributed specifically to P-32 implantation or interpreted as evidence of a synergistic effect. Third, the 16-week LDCR was assessed over a short interval and included stable disease as well as objective responses. The 100% estimate was based on 16 evaluable patients, whereas the corresponding all-treated estimate was 94.1%; both estimates remain imprecise because of the small denominator and should not be interpreted as evidence of therapeutic efficacy. Fourth, the four patients who underwent surgery constituted a selected subgroup identified after multidisciplinary reassessment, and the observed resection rate cannot establish that P-32 increased conversion to surgery. Finally, the OS analysis was based on only three deaths, substantial censoring, and a median follow-up of 15.6 months. The Kaplan–Meier-derived median OS estimates are therefore statistically immature and highly unstable. Moreover, survival calculated from chemotherapy initiation reflects the overall combined treatment pathway and cannot isolate any contribution from P-32 implantation. The small cohort also limits the ability to identify uncommon procedure-related adverse events and to distinguish toxicities related to implantation from those associated with chemotherapy. Accordingly, this study should be regarded as a preliminary feasibility and safety study, and all tumour-response, resection, and survival outcomes should be considered descriptive and hypothesis-generating.

Despite these limitations, the present cohort provides preliminary real-world data regarding the technical feasibility, safety, and early local disease-control outcomes of intratumoral P-32 microparticle implantation. Given the small sample size and absence of a comparator group, these findings should not be interpreted as evidence of therapeutic efficacy. In contrast to controlled trial settings, real-world cohorts capture variability in patient selection, clinical decision-making, and follow-up, and may therefore complement prospective data by demonstrating reproducibility of early signals in routine practice [33,34]. The eligibility criteria and technical requirements for EUS-guided implantation resulted in a highly selected patient population, predominantly comprising individuals with good performance status, limited tumour size, preserved organ function, and favourable anatomy for implantation. This may have introduced selection bias and may limit the generalizability of the findings to the broader population of patients with LAPC, particularly those with poorer performance status, larger or anatomically complex tumours, greater comorbidity, or technically inaccessible lesions.

Future analyses should include a larger cohort and longer follow-up. Integration of this locoregional approach with contemporary systemic therapy regimens and exploration of its role as a bridge to surgical resection warrant further investigation. Collectively, these findings support further prospective investigation of intratumoral radioactive microparticle therapy as a locoregional strategy for pancreatic cancer.

## 5. Conclusions

In this prospective single-center pilot study, EUS-guided intratumoral P-32 microparticle implantation combined with gemcitabine plus nab-paclitaxel was technically feasible in a highly selected group of patients with unresectable LAPC. No implantation-related procedural complications were recorded, and the observed adverse events were predominantly low-grade, providing preliminary safety information. However, the small sample size, absence of a chemotherapy-only comparator, selected patient population, and limited follow-up preclude conclusions regarding therapeutic efficacy. The observed local disease control, tumour and CA19-9 reductions, subsequent resections, and exploratory survival estimates describe outcomes of the combined treatment strategy but cannot be attributed specifically to P-32 implantation. These findings should therefore be considered hypothesis-generating and warrant confirmation in adequately powered controlled studies with broader patient populations and longer follow-up.

## Figures and Tables

**Table 1:** Baseline characteristics and treatment delivery.

Characteristic	Value
Patients included, n	17
Age, years, median (IQR)	70 (60–75)
**Sex, n (%)**	
Male	11 (64.7)
Female	6 (35.3)
**ECOG performance status, n (%)**	
0	10 (58.8)
1	7 (41.2)
**Primary tumour location, n (%)**	
Pancreatic head	11 (64.7)
Pancreatic body	4 (23.5)
Pancreatic neck	1 (5.9)
Uncinate process	1 (5.9)
Tumour volume, cc, median (IQR)	20.0 (11.6–29.0)
OncoSil volume, mL, median (IQR)	1.6 (0.9–2.3)
Administered activity, MBq, median (IQR)	10.5 (6.1–15.2)

## Data Availability

The data that support the findings of this study are available from the corresponding author upon reasonable request. The data are not publicly available due to privacy and ethical restrictions.
